# Pharmacologic rationale, efficacy and safety of the fixed-dose co-formulation of indacaterol and glycopyrronium

**DOI:** 10.1186/2049-6958-9-64

**Published:** 2014-12-08

**Authors:** Girolamo Pelaia, Rosario Maselli, Luca Gallelli

**Affiliations:** Department of Medical and Surgical Sciences, Section of Respiratory Diseases, University “Magna Graecia” of Catanzaro, Campus Universitario “S. Venuta”, Viale Europa, Località, Germaneto, 88100 Catanzaro, Italy; Department of Health Science, Section of Pharmacology, University “Magna Graecia” of Catanzaro, Catanzaro, Italy

**Keywords:** Co-formulation, Dual bronchodilation, Glycopyrronium, Indacaterol, LABA, LAMA, Synergism, QVA149

## Abstract

Chronic obstructive pulmonary disease (COPD) is a widespread respiratory disorder, usually characterized by progressive and poorly reversible airflow limitation. Inhaled long-acting bronchodilators, namely LABA (long-acting β_2_-adrenergic agonists) and LAMA (long-acting muscarinic receptor antagonists) are the mainstay of COPD treatment. Because the symptoms of many patients with COPD do not satisfactorily improve by using a single, either LABA or LAMA bronchodilator, the synergism of action resulting from the combination of the different bronchodilating mechanisms activated by LABA and LAMA, respectively, can significantly contribute to a better disease control. Based on these clinical and pharmacological considerations, several LABA/LAMA fixed-dose combinations have been developed and experimentally evaluated. Within such a context, the drug co-formulation containing indacaterol and glycopyrronium is probably the LABA/LAMA association which has been most extensively studied during the last few years.

## Introduction

COPD is characterized by a barely reversible airflow limitation, thus it is easily understandable that inhaled bronchodilators such as LABA and LAMA are the main pharmacological options in order to improve lung function, dyspnea, exercise tolerance and overall quality of life, as well as to prevent disease exacerbations [1]. Moreover, current guidelines recommend the combined use of LABA and LAMA when symptoms are not adequately improved by a single bronchodilator [2]. The rationale of using these two drug classes is based on their ability to effectively interfere with the bronchoconstrictive signaling network leading to the respiratory dysfunction underlying COPD. In particular, by means of different mechanisms of action, LABA and LAMA can counteract the increased bronchomotor tone, which in COPD patients is mainly sustained by an excessive activation of cholinergic pathways. Therefore, LABA and LAMA act cooperatively by reciprocally potentiating their bronchodilating effects [3]. These positive pharmacological interactions can be further amplified by the different distribution patterns of receptor targets throughout the bronchial tree. Indeed, bronchodilating β_2_-adrenergic receptors (β_2_-ARs) are located along all the respiratory system, but their density progressively increases from the proximal, larger airways towards the distal, smaller ones. Vagal cholinergic innervation is mostly distributed within the central airways, where bronchoconstrictive muscarinic receptors are present in higher numbers; however, extraneuronal acetylcholine (ACh) mainly activates muscarinic receptors placed in peripheral airways [4]. Among LABA/LAMA combinations, a remarkable amount of pharmacological and clinical information has been obtained by evaluating the effects of the fixed-dose co-formulation of indacaterol and glycopyrronium, known as QVA149 [5]. Indacaterol is a LABA used once daily, as it provides a prolonged bronchodilation, lasting at least 24 hours [6, 7]. Glycopyrronium (NVA237) is a recently developed LAMA that, similarly to indacaterol, induces a persistent, 24 hour-long bronchodilation, thus being also suitable for once daily administration [8, 9]. Therefore, by acting via their distinct mechanisms of action, when used together indacaterol and glycopyrronium can exert synergistic effects, thereby optimizing and maximizing bronchodilation in those COPD patients whose needs are not adequately met by a monotherapy performed with a single bronchodilator.

The aim of this review article is firstly to outline the mechanisms of action of QVA149, and then discuss its clinical and functional effects, as well as the safety and tolerability profile.

## Review

### Basic mechanisms of action

**Figure 1:**
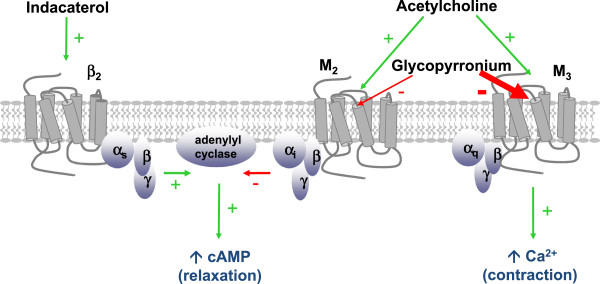
**Dual bronchodilation induced by the fixed-dose co-formulation of indacaterol/glycopyrronium.** Indacaterol induces a long-lasting relaxation of airway smooth muscle via a prolonged activation of β_2_-adrenergic receptors, coupled through a stimulatory G protein (consisting of α_s_, β and γ subunits) to adenylyl cyclase, which catalyzes the synthesis of the intracellular second messenger cAMP. The bronchodilation induced by indacaterol is greatly enhanced by glycopyrronium, which provides a prolonged competitive blockade of M_3_ muscarinic receptors. Indeed, glycopyrronium dissociates very slowly from these receptors, whereas it quickly detaches from M_2_ receptors. Although the latter may partially contribute to bronchoconstriction via inactivation of adenylyl cyclase mediated by an inhibitory G protein (consisting of α_i_, β and γ subunits), they are, however, primarily responsible for pre-junctional inhibition of acetylcholine release.

All β2-adrenergic receptor (β_2_-AR) agonists, also including indacaterol, relax airway smooth muscle (ASM) regardless of the nature and multitude of bronchoconstrictive stimuli, thus acting as functional antagonists of bronchoconstriction. In particular, these drugs activate β_2_-ARs, which are coupled to the stimulatory G protein (Gs) that is, in turn, responsible for the stimulation of adenylyl cyclase (AC) and the subsequent increase in the concentration of intracellular second messenger cyclic adenosine 3’5’-monophosphate (cAMP) (Figure 1) [10]. The latter activates cAMP-dependent protein kinase A (PKA), which phosphorylates several targets within the cell, thereby leading to inhibition of Ca^2+^-dependent ASM contraction [11]. Indacaterol is a powerful β_2_-adrenergic agonist, being able to induce a very fast and prolonged bronchodilation, which lasts for approximately 24 hours [12]. This drug has an intense intrinsic activity at the level of β_2_-ARs, and its bronchodilating action does not elicit significant tachyphylaxis. Unlike salmeterol, indacaterol does not alter the fluidity of cell membrane, and its long-lasting bronchodilating effect is due to a high affinity of the lipophilic tail of the molecule for the so-called “lipid rafts” [13]. These consist of micro-domains rich in cholesterol and sphingolipids, that**,** within the cell membrane, function as aggregation sites for β_2_-ARs, thus also facilitating their connection with the signaling pathway including Gs and AC. The rapid onset of the bronchodilating action of indacaterol depends on its hydrophilic head, which interacts with the β_2_-AR hydrophilic pocket surrounded by the seven hydrophobic transmembrane domains of this G protein-coupled receptor.

The bronchoconstrictive effect of ACh can be inhibited by inhaled anticholinergic drugs, which act through a competitive antagonism of muscarinic receptors. Glycopyrronium bromide is a powerful once-daily inhaled anticholinergic agent, characterized by a high kinetic selectivity for M_3_ versus M_2_ muscarinic receptors (Figure 1) [14, 15]. Therefore, glycopyrronium operates via a prolonged blockade of M_3_ receptors, whereas this drug rapidly dissociates from M_2_ receptors. With regard to airway muscarinic receptors, this kinetic pattern of glycopyrronium is very relevant, in that ACh-dependent ASM contraction is mainly mediated by M_3_ receptors, whilst M_2_ receptors are primarily responsible for prejunctional inhibition of ACh release from postganglionic vagal nerves [16, 17]. Furthermore, cardiac M_2_ receptors modulate atrial pacemaker activity, atrio-ventricular conduction and ventricular electro-mechanical coupling [18]. A persistent blockade of M_2_ muscarinic receptors could thus attenuate bronchodilation primarily due to M_3_ receptor antagonism, as well as increase the risk of unwanted cardiovascular side effects, including tachycardia and arrhythmias.Therefore, by largely preserving M_2_ receptor functions, glycopyrronium maximizes bronchodilation and is also characterized by a very good pattern of cardiovascular safety [8, 9].

The remarkable bronchodilating action of glycopyrronium is thus mainly mediated by a very effective prevention of the activation of M_3_ receptors, coupled to the Gq protein that, in turn, stimulates the β isoform of phospholipase C (PLC-β), which cleaves phosphatidyl-inositol 4,5-bisphosphate (PIP_2_) into two intracellular second messengers: inositol 1,4,5-trisphosphate (IP_3_) and 1,2 diacylglycerol (DAG) [19]. IP_3_ activates its receptor localized in the sarcoplasmic reticulum (SR), thereby releasing into the cytosol calcium ions (Ca^2+^) which bind to calmodulin, thus inducing the activation of myosin light chain kinase (MLCK). MLCK phosphorylates myosin light chain (MLC), thereby promoting the subsequent actin-myosin interaction, which is responsible for ASM contraction. The long-lasting bronchodilating effect of glycopyrronium is associated with a very fast onset of action, due to the rapid occupancy of muscarinic receptors. Such an immediate blockade of ASM M_3_ receptors is thus responsible for a prompt decrease in cytosolic Ca^2+^ levels, paralleled by a rapid inhibition of bronchoconstriction elicited by cholinergic agents [15]. The advantageous bronchodilating pattern of glycopyrronium explains its excellent therapeutic profile, emerging from several clinical trials. In particular, the GLOW1 and GLOW2 studies showed that glycopyrronium provides a rapid and sustained FEV_1_ (forced expiratory volume in one second) increase, already detectable within 5 minutes of administration [9, 20].

In addition to their direct bronchodilating effects, LABA and LAMA are also capable of indirectly affecting bronchomotor tone by implementing, inside ASM, important cross-talks between cholinergic and adrenergic pathways. Hence, by powerfully stimulating β_2_-ARs, indacaterol can inactivate the signaling cascade underlying ACh-induced bronchoconstriction via an effective inhibition of the activity of M_3_ muscarinic receptor-coupled Gq protein. This mechanism is mediated by a cAMP/PKA-dependent transcriptional stimulation of the expression of regulator of G-protein signaling 2 (RGS2), which specifically inhibits Gq activation [21]. On the other hand, muscarinic receptor antagonists such as glycopyrronium can either restore or potentiate and amplify the bronchodilating action of β_2_-adrenergic agonists not only by inhibiting the IP_3_-dependent intracellular effects triggered by M_3_ receptor stimulation, but also by preventing the deleterious effects on β_2_-AR function elicited by the ACh-activated M_3_/Gq/PLC/DAG/PKC signaling pathway. Indeed, by stimulating ASM M_3_ receptors, ACh is also able to induce DAG-mediated activation of protein kinase C (PKC) [19]. PKC can thus phosphorylate specific serine residues located within the third intracellular loop of β_2_-AR, as well as the α subunit of Gs protein, thereby uncoupling these two key components of the signal transduction cascade underlying the bronchodilating action of β_2_-adrenergic agonists [22, 23]. Moreover, PKC can also phosphorylate and activate G-protein coupled receptor kinase 2 (GRK2), which in turn promotes the phosphorylation of specific threonine and serine residues located within the C-terminal cytoplasmic tail of β_2_-AR, thus further enhancing β_2_-AR/Gs uncoupling as well as facilitating binding of β-arrestins to the C-terminus of β_2_-AR, with subsequent intracellular receptor sequestration [24, 25].

The above mentioned cooperation between indacaterol and glycopyrronium, occurring within ASM at a postjunctional level, can also extend to the pre-junctional site, i.e. at the level of vagal postganglionic nerve endings. Prejunctional inhibition of ACh release is indeed mediated by indacaterol-dependent β_2_-AR activation, as well as by stimulation of M_2_ muscarinic inhibitory receptors [3]. Indeed, the function of prejunctional M_2_ receptors is largely preserved by glycopyrronium, which dissociates very rapidly from them, whereas this drug blocks for a long time the postjunctional M_3_ muscarinic receptors (Figure 1), mainly responsible for bronchoconstriction and mucus hypersecretion.

### Clinical and functional effects

On the basis of the above discussed pharmacological concepts, several controlled trials have been carried out to evaluate the potential benefits of QVA149 in COPD treatment [5]. The positive results of such studies have led to the recent approval of this drug co-formulation in Europe, Canada and Japan [26–28]. All these trials have been performed using the Breezhaler® dry powder device, containing a capsule including 110 mcg of indacaterol and 50 mcg of glycopyrronium. The delivered dose, leaving the inhaler at the level of its mouthpiece, consists of 85 mcg of indacaterol and 43 mcg of glycopyrronium, respectively.

The double-blind, randomized and placebo-controlled trial called ENLIGHTEN was carried out for 52 weeks in 339 patients with moderate-to-severe COPD, who exhibited post-bronchodilator FEV_1_ measurements ranging from 30% to 80% of predicted values [29]. QVA149 significantly improved lung function, thus steadily increasing FEV_1_ throughout the whole study period. Furthermore, QVA149 was remarkably more effective than placebo in decreasing both respiratory symptoms and the use of as-needed short-acting rescue bronchodilators [29]. The main aim of the SPARK study, performed in 362 centers located in 27 countries, was to evaluate the efficacy of QVA149, by comparing it over 64 weeks to either glycopyrronium or tiotropium, with regard to prevention of COPD exacerbations [30]. In particular, 2,224 patients with severe or very severe COPD were enrolled, presenting a post-bronchodilator FEV_1_ less than 50% of predicted, who had experienced at least one exacerbation in the year prior to enrollment. Therefore, following a double-blind, randomized design, 741 subjects were assigned to treatment with QVA149, 741 to monotherapy with glycopyrronium (50 mcg daily through Breezhaler device) and 742 to monotherapy with tiotropium (18 mcg daily via HandiHaler device). The results showed that QVA149 was able to significantly reduce the overall rate of all COPD exacerbations (mild, moderate and severe), at rates of 15% and 14% when compared to glycopyrronium and tiotropium, respectively [30]. Furthermore, a recent meta-analysis demonstrated that QVA149 can delay by 35% the onset of the next exacerbation [31]. With respect to glycopyrronium and tiotropium, the SPARK study also showed that QVA149 was able to induce a persistently and significantly greater increase in trough FEV_1_. Moreover, QVA149 was also more effective than either glycopyrronium or tiotropium in improving the global health status, as assessed by St. George’s Respiratory Questionnaire (SGRQ).

In the SHINE study, 2,144 patients with moderate-to-severe COPD (post-bronchodilator FEV_1_ range: from 30% to 80% of predicted values) were randomly divided into five groups, each one receiving for 26 weeks, as once daily administration, one of the following treatments: 1) QVA149 (475 patients); 2) indacaterol 150 mcg (477 patients), 3) glycopyrronium 50 mcg (475 patients); 4) tiotropium 18 mcg (483 patients); 5) placebo (234 patients) [32]. With the exception of the group of patients undergoing treatment with tiotropium, who used the dry powder inhaler “HandiHaler”, all the other groups used Breezhaler as dry powder device. The majority of patients were male (75.4%), and had not experienced exacerbations during the year prior to enrollment (74.6%). Before starting the various treatments, there were no significant spirometric differences among the five groups. The results of the spirometries performed at week 26 of treatment showed that QVA149 induced, when compared to monotherapies with either indacaterol, glycopyrronium, tiotropium or placebo, significantly greater increases in trough FEV_1_ and peak expiratory flow (PEF) [32]. With respect to placebo, tiotropium and glycopyrronium at week 12, as well as in comparison to tiotropium and placebo at week 26, QVA149 induced a greater improvement in dyspnea, assessed by transition dyspnea index (TDI). At the end of the study the overall health status, evaluated through the SGRQ score, resulted to be also significantly improved in the group treated with QVA149, when compared to the groups treated with either tiotropium or placebo. Furthermore, a significant decrease in the consumption of as needed short-acting bronchodilators, used as rescue medications, was detected in the group treated with QVA149, when compared to the other four groups.

The aim of the multicenter, double-blind, randomized ILLUMINATE trial, was to assess the effectiveness of QVA149 in comparison to the fixed LABA/ICS (inhaled corticosteroid) combination consisting of salmeterol 50 mcg plus fluticasone propionate 500 mcg (SFC), delivered twice daily via Diskus dry powder inhaler [33]. In particular, this study lasted 26 weeks and included 523 current or ex-smokers with moderate to severe COPD (age ≥ 40 years), who had no disease exacerbation during the year prior to their enrollment. Airflow limitation was characterized by a post-bronchodilator FEV_1_ ranging from 40% to 80% of the predicted values. The results of this study showed that QVA149 induced, when compared to SFC, a significantly higher FEV_1_ increase, already detectable during the first study day, as well as at the 12^th^ and 26^th^ week [33]. QVA149 was significantly more effective than SFC in decreasing the use of as needed short-acting bronchodilators, as well as in improving dyspnea, assessed by TDI. No significant difference was found between the two treatments with regard to health status, assessed by SGRQ [33]. A logical extension of this study is the ongoing FLAME trial, which is investigating the effects of QVA149, compared with SFC, on COPD exacerbation rates [34]. LANTERN is a further ongoing study which is evaluating the efficacy of QVA149 versus SFC over 26 weeks, in COPD patients from Argentina, Brazil, Chile, China and Taiwan [35].

The BRIGHT study investigated the effects of QVA149 on exercise tolerance, lung volumes, and dynamic hyperinflation in patients with moderate-to-severe COPD over 3 weeks [36]. This trial showed that exercise endurance time was significantly increased by QVA149 when compared to placebo, with an improvement pattern similar to that induced by tiotropium. Moreover, in comparison to placebo and tiotropium, QVA149 elicited significantly greater decreases in lung hyperinflation both at rest and during exercise [36]. Of remarkable clinical relevance are also the results of the BLAZE study, a multicentre, blinded, double-dummy, crossover trial, which enrolled 247 patients with moderate-to-severe COPD who were randomly assigned to receive once-daily QVA149, placebo or tiotropium [37]. Improvements in dyspnea were assessed via the self-administered computerized (SAC) version of TDI after 6 weeks. At this time, the SAC TDI total score resulted significantly higher when patients used QVA149, as compared with both placebo and tiotropium. Treatment with QVA149 was also associated, when compared to either placebo or tiotropium, with a significantly lower use of rescue medications [37].

Furthermore, several studies showed that, in addition to the excellent medium- and long-term clinical and functional effects induced by QVA149, the therapeutic profile of this drug co-formulation is also characterized by a very fast onset of bronchodilation, already detectable at 5 min post-dose on day one [32, 33, 37, 38]. This feature of QVA149 is very important in that it makes possible to provide an immediate symptom relief, especially in the morning soon after awakening. Overall, the efficacy of QVA149 is very similar to that achievable with the concurrent administration of its monocomponents indacaterol and glycopyrronium [39]. Indeed, the BEACON trial, which enrolled 193 patients randomized to receive either QVA149 or indacaterol plus glycopyrronium as concurrent, independent inhalations, documented that both treatment groups experienced similar FEV_1_ improvements, as well as analogous reductions in symptom scores and rescue medication use [39].

### Safety and tolerability profile

According to many studies, QVA149 is well tolerated and is characterized by a favourable safety profile with respect to its individual monocomponents [26]. A pooled analysis of 6-month safety data extrapolated from SHINE, ILLUMINATE, ENLIGHTEN and ARISE (a safety study carried out in Japanese patients) trials, showed that the proportion of subjects treated with QVA149 who experienced cardio- and cerebro-vascular events was very similar to that detected in COPD patients treated with tiotropium (1.8% and 1.7%, respectively), and lower than that observed in patients receiving treatment with placebo (2.6%) [40]. The proportion of patients who reported one or more cardio- and cerebro-vascular serious adverse events in the QVA149 group was 0.6%, comparable to or slightly lower than those reported by patients undergoing other active treatments [40]. Additionally, no significant differences among QVA149 and the other treatment groups were detected with regard to the incidence of unwanted side effects in SPARK, BRIGHT and BLAZE studies [30, 36, 37].

In general, once-daily dosing of QVA149 is well accepted by COPD patients [5]. Moreover, the combined use of indacaterol and glycopyrronium is further favoured by the inhalation device used. In fact, the dry powder inhaler Breezhaler is characterized by a low airflow resistance, that thus allows the activation of the device with an affordable inspiratory effort [41]. This feature is very important because Breezhaler can thus be easily used even by patients with the most severe forms of COPD, which of course include the main candidates for the dual, LABA/LAMA, combined therapy. In fact, the magnitude of the peak inspiratory flow (PIF) decreases gradually as disease severity increases. However, since Breezhaler can be activated by a relatively low inspiratory flow, its use is markedly suitable for almost all patients with COPD, also including those characterized by the most severe degrees of respiratory functional impairment. In addition, Breezhaler has other remarkable advantages, especially related to auditory, gustatory and visual perceptions, that make it possible for the patient to be sure of having properly inhaled drug medications. Indeed, the release of a drug fixed dose by Breezhaler is associated with an audible hum in the inhalation chamber, the sensation of a sweet taste due to the presence of lactose, and the appreciation of powder emptying due to the transparency of the capsule inserted into the inhaler. All these features of Breezhaler, associated with the advantage of once daily administration, guarantee a high degree of compliance by COPD patients, with a consequent considerable increase in the adherence to prescribed inhaled therapy [42].

## Conclusions

Based on the above study results, it can thus be argued that a dual bronchodilating treatment, performed through the use of an inhaled pharmaceutical formulation capable of simultaneously delivering indacaterol and glycopyrronium, is characterized by a greater therapeutic effectiveness when compared to monotherapies consisting of each drug administered alone. Therefore, this experimental evidence confirms the pharmacologic rationale underlying the synergistic potentiation of the bronchodilating actions exerted by different drugs such as LABA and LAMA, which act via distinct mechanisms of action. Such an enhanced therapeutic efficacy can be achieved without increasing the incidence of adverse events. This suggests that the co-formulation of indacaterol and glycopyrronium can be usefully utilized to optimize and maximize bronchodilation in many COPD patients, who do not experience an adequate airflow increase by using a single bronchodilator. This results in greater improvements in subjective symptoms, lung function and overall quality of life. In addition, the enhancement of airway stabilization and the long-term persistence of airway patency (pharmacologic “airway stenting”), associated with a more effective prevention of COPD exacerbations, are likely to slow the rate of disease progression, as well as to concomitantly reduce mortality.
